# Utilization of Polypropylene in the Production of Metal-Filled Polymer Composites: Development and Characteristics

**DOI:** 10.3390/ma13122856

**Published:** 2020-06-25

**Authors:** Volodymyr Moravskyi, Anastasiia Kucherenko, Marta Kuznetsova, Ludmila Dulebova, Emil Spišák, Janka Majerníková

**Affiliations:** 1 Department of Chemical Technology of Plastics Processing, Lviv Polytechnic National University, 12 St. Bandera str., 79013 Lviv, Ukraine; vmoravsky@gmail.com (V.M.); anastasiyakucherenko05@gmail.com (A.K.); 2 Department of Heat Engineering and Thermal and Nuclear Power Plants, Lviv Polytechnic National University, 12 St. Bandera str., 79013 Lviv, Ukraine; kuznetsovam83@gmail.com; 3 Department of Engineering Technologies and Materials, Faculty of Mechanical Engineering, Technical University of Košice, Mäsiarska 74, 04001 Košice, Slovakia; emil.spisak@tuke.sk (E.S.); janka.majernikova@tuke.sk (J.M.)

**Keywords:** polypropylene, polymer waste, metallization, chemical recovery, copper, metal filled polymer composite

## Abstract

Metal-filled composites based on polypropylene waste have been successfully obtained with an injection molding method of metalized polymer raw materials. Using the model polymer, the peculiarities of the formation of the copper layer in solutions of chemical metallization on the polypropylene surface were investigated and the main factors influencing this process were established. The main influence on the rate of reduction of copper in solutions of chemical metallization has the concentration of copper sulfate, sodium hydroxide, and EDTA-Na_2_. It was shown that the efficiency of the copper plating process also strongly depends on polymer processing, which follows the activation. In case of the use of simple activation, it is not possible to obtain metalized raw materials with high efficiency. Additional processing of activated polymer raw materials is required to carry out the process with high efficiency. The amount of reduced copper on the polymer surface can be adjusted by changing the concentration of the components of the chemical metallization solution, as well as the degree of loading of the polymer raw material. Examination by electron scanning microscopy of the obtained metalized polypropylene showed that the copper coating on the polymer particles is formed with a high degree of surface coverage. The formed copper coating is free of copper oxides, which is confirmed by X-ray diffraction studies and analysis of the spectrum of characteristic X-rays. Metal-filled composites have been characterized by the effect of copper on mechanical and rheological (MFR) properties. The Differential Scanning Calorimetry (DSC) and Thermogravimetric (TG) methods show a certain effect of metal on the magnitude of thermal effects and the rate of weight loss.

## 1. Introduction

Significant interest of the modern industry in the use of polymer composite materials is caused by a set of valuable properties that characterize these materials. In many cases, they are a real alternative to traditional materials, for example in the manufacture of products for structural purposes [1,2,3]. Moreover, new special-purpose materials can be obtained on their basis [4,5,6,7,8,9,10].

The widespread introduction of polymer composite materials in various fields of science and technology is also facilitated by the fact that the technology of their production has almost unlimited possibilities to change their properties. The combination of both the polymer matrix and the filler properties can provide new materials with a set of necessary properties, as well as predict these properties at the stage of obtaining the material [11,12,13,14,15,16,17,18,19,20].

When creating polymer composite materials for special purposes, the use of high-tonnage thermoplastics as a polymer matrix and metal fillers is promising. Such a combination allows us to obtain materials with the required operational, physicomechanical, and physicochemical properties, as well as a relatively low cost. Besides, the use of waste products and waste materials as polymer raw materials will contribute to even greater cost reduction, and most importantly, a solution to the number of acute environmental and socio-economic problems. This will also expand their possible recycling and reuse in the form of high-tech metal-filled polymer composites [21,22,23].

Metal-filled polymer composites can be used as antifriction, heat-conducting, antistatic, and shielding materials [24,25], as well as act as a basis for the creation of highly efficient heat storage systems, in which due to the heat of the phase transition it is possible to accumulate a significant amount of heat and reduce the drawback of conventional materials with a phase transition-low thermal conductivity [26,27,28,29,30]. Highly crystalline and high-tonnage polymers such as polyethylene, polypropylene, polyamide, polyethylene terephthalate, etc. are promising in this regard.

In the simplest case, the metal-filled polymer composite consists of metal particles that are evenly distributed in the polymer matrix [31]. The main disadvantage of this system is that at low concentrations of filler, they remain isolated from each other and do not contribute to the conductivity of the system. The mechanical properties of the system deteriorate sharply with the next increased concentration [32]. Thus, the creation of polymer composites that combine good conductive (electrical and thermal) and mechanical properties is a difficult task and is of considerable practical interest.

Obtaining metal-filled composite materials with high technological and operational properties requires the development of alternative technological solutions for their production. The technology of obtaining metal-filled polymer composites by the metallization of polymer raw materials with its subsequent processing by standard methods directly into products was used [33,34,35,36]. As a result, the process of combining the components is significantly facilitated and uniform distribution of the metal filler in the polymer matrix is ensured. This technology is a highly efficient, resource-saving technological process and is characterized by a shortened production cycle.

The technology is based on the use of mechanical activation of the polymer surface in order to give it catalytic activity before the deposition of metal in chemical reduction solutions. The use of such an activation technology allows us to avoid the main disadvantages of classical metallization technology: a large number of preliminary operations to prepare the polymer surface to give it catalytic activity and the use of hazardous and expensive reagents [37]. The process of chemical activation of the polymer surface was simplified as a result of simultaneous processing in a ball mill of a polymeric material with a powdered metal activator. As a result of such processing, the metal activator is firmly fixed on the polymer surface and provides the polymer surface with the catalytic activity required for the formation of the base layer of the metal in chemical reduction solutions [38]. The effectiveness of the method of mechanical activation is determined by at least two factors: reduction of the number of technological operations and reduction of the number of expensive and harmful chemicals.

The main tasks that the researchers face in the development of new technological processes is to establish the main factors that affect this process. Information about the process patterns allows it to be carried out in controlled conditions, which guarantees obtaining the products of the required quality with minimal resource costs. This study is aimed at establishing the patterns of the process of copper plating of the polymer surface and obtaining metallized polymeric raw materials, which is the first step in developing an effective technology for obtaining metal-filled composites. Information about the patterns of formation of the metal layer on the polymer surface is essential for the possibility of identifying the main factors that will ensure the process in controlled conditions.

## 2. Experimental

### 2.1. Materials and Obtaining of Metalized Polymeric Raw Materials

**Figure 1:**
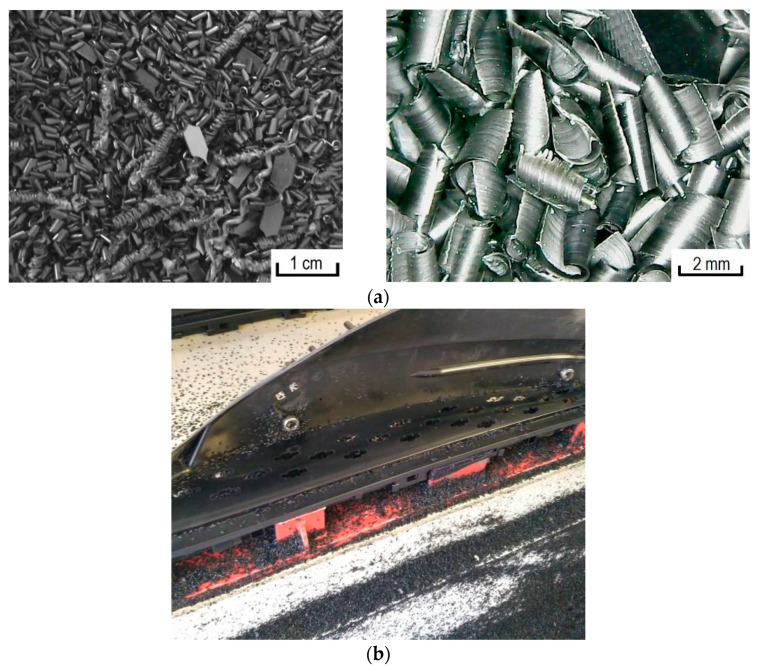
Polypropylene waste (**a**) obtained by milling panels (**b**) for forming cutouts.

Two types of polypropylene (PP) were used as a polymer matrix to study the peculiarities of metallization and production of metal-filled composites: polypropylene brand Moplen HF501N (LyondellBasell, Houston, TX, USA) and polypropylene waste (Figure 1a), which is obtained as a result of mechanical processing of car interior panels. This processing was carried out to ensure the controlled destruction of the panels and was performed at the Technical University of Kosice (Kosice, Slovakia) (Figure 1b).

Zinc powder of super extra fraction (Norzinco GmbH, Goslar, Germany) was used as a metal activator. Activation of the polypropylene surface was performed in a laboratory ball mill with a volume of 4 liters with ceramic cylindrical grinding bodies, rotation speed is 100 rpm. The mill was loaded with polymer and zinc powder, the ratio of polypropylene: Zn powder was 50:9 wt.%, processing time 1 h. During the rotation of the mill, the activator metal was fixed on the polymer surface.

**Figure 2:**
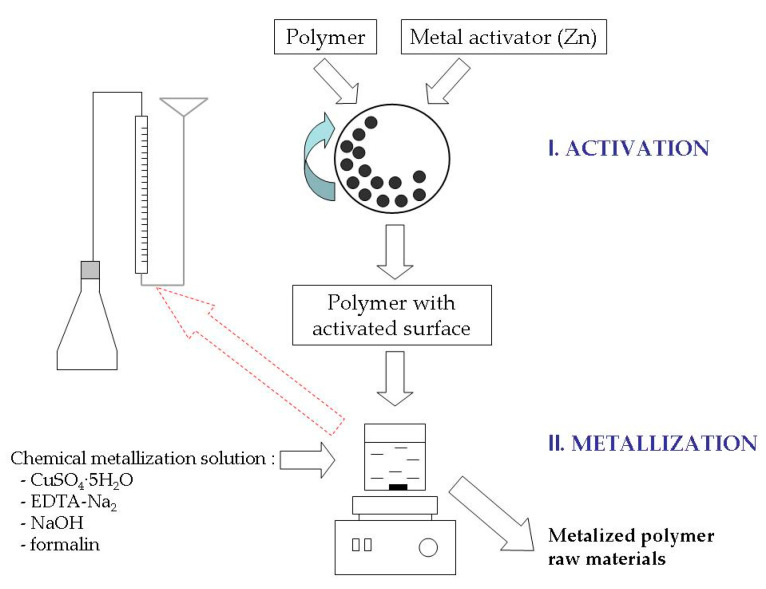
Scheme of obtaining metalized polymeric raw materials.

The next stage is the formation of a metal layer on the activated polypropylene surface as a result of the reduction of copper ions in chemical metallization solutions (Figure 2). Solutions of the following composition were used for metallization: CuSO_4_∙5H_2_O brand “pure for analysis”, EDTA-Na_2_ (C_10_H_14_N_2_Na_2_O_8_∙2H_2_O) brand “pure for analysis”, NaOH brand “pure”, formalin stabilized [33,35,37]. Concentrations of components of chemical metallization solutions were within (mmol/L): CuSO_4_∙5H_2_O-48–144, EDTA-Na_2_-47–67, NaOH-250–560, formalin 366. The concentration of formalin was constant and was not considered as a factor that influenced the process. The metallization was carried out with vigorous stirring using a magnetic stirrer, the volume of the chemical metallization solution in all cases was 200 mL.

### 2.2. Kinetics of Metallization

Studies of the kinetics of copper layer formation in chemical metallization solutions were performed by the volumetric method. The volumetric method of studying the kinetics of metallization of the activated polymer surface is based on the peculiarity of the reduction of copper ions in solutions with the complexing agent EDTA-Na_2_. In such solutions, one mole of hydrogen is released per mole of reduced copper, the volume of which is measured [31,33]. To do this, the metallization was performed in an airtight container, which was connected to a measuring tube where water was displaced by gas (hydrogen). To illustrate the results of volumetric studies, the average values of at least 5 studies are presented. The average deviation between the results of the research is not more than 5%.

### 2.3. Sieve Analysis and Calculation of the Polymer Surface Area

Sieve analysis was used to characterize the particle size distribution of polypropylene brand Moplen HF501N.

The average particle radius (mm) of polypropylene on a certain sieve was calculated from the function:

(1) $$d_{aV}=\frac{r_{n-1}+r_{n}}{2}$$

where, $r_{n-1}$—the size of the previous sieve cell, $r_{n}$—sieve cell size.

The surface area of a certain fraction of polypropylene was calculated based on the following considerations:

1. The surface area of one particle was calculated by using the average particle diameter of polypropylene on a certain sieve:

(2) $$S_{on}=\pi \times d_{aV}^{2}$$

2. To calculate the total area, the number of particles of a certain mass is needed, so:

2.1. the total volume (monolith) of the polymer should be calculated:

(3) $$V_{t}=\frac{m}{\rho }$$

where m—the mass of the polymer, ρ—density of polypropylene brand Moplen HF501N (900 kg/m^3^);

2.2. the volume of one particle of polypropylene:

(4) $$V_{on}=\frac{4}{3}\times \pi \times r^{3}$$

where r—the average radius of the particle;

2.3. the number of particles:

(5) $$n-\frac{V_{t}}{V_{on}}$$

3. The total area of the particles:

(6) $$S_{t}=S_{on}\times n$$

### 2.4. Calculation of Metal Content and Metallization Efficiency

To calculate the copper (zinc) content of the metalized (activated) polypropylene raw material, it was weighed to the nearest 0.0005 g, treated with 50% nitric acid and, after filtration, washing and drying to constant weight, weighed again. The metal content was calculated by the formula:

(7) $$X=\frac{m-m_{1}}{m}\times 100$$

where m—a mass of metalized (activated) polymer, $m_{1}$—the mass of the polymer after etching, washing, and drying.

The metallization efficiency (in%) was calculated by the ratio of the mass of copper on polypropylene after metallization to the theoretical mass of copper, which can be formed by the reduction of all copper ions that were introduced into the solutions of chemical metallization.

### 2.5. Obtaining a Metal-Filled Polymer Composite

Obtaining a metal-filled polymer composite was the result of processing of metallized polypropylene by injection molding. When obtaining products from metallized polypropylene by injection molding, the melting of polypropylene and its flow appear. This destroys the layer of metal that covers the polypropylene particles and the metal is evenly distributed in the polymer matrix during the production of the product (in our case, samples for physical and mechanical studies).

### 2.6. Test Methods

The mechanical properties of composites in tension were investigated according to ISO 527-5: 2009, using the samples of type 1B. Tensile tests were performed using a universal testing machine Zwick/Roell Z010 (Zwick/Roell, Ulm, Germany). Five samples were used for each composite composition. Tensile tests were performed at a speed of 5 mm/min. Samples were obtained on a Demag Ergotech pro 25-80 machine (Wiehe, Germany). The temperature in the zones of the material cylinder was 190, 220, 240 °C. The temperature of the mold was 20 °C. The holding under pressure time was 10 s, and the cooling time was 10 s. The injection pressure was 80 MPa. A stationary cold channel two-slot mold was used.

The rheological properties of metalized polymer raw materials were characterized by value MFR on the device IIRT-AM (“ASMA-Pribor”, Svitlovodsk, Ukraine) at a temperature of 190 and 230 °C and loaded with 10 and 5 kg, respectively.

Differential scanning calorimetry and thermogravimetric analysis were performed for the polymer raw material and metal-filled composite using the SDT Q 600 (TA Instruments, New Castle, PA, USA) in an inert (argon) atmosphere. The heating rate of the samples is 10 K/min.

Microscopic examinations were performed using an optical microscope SIGETA Expert 10–300× 5.0 Mpx (SIGETA, Kiev, Ukraine) and scanning electron microscope-microanalyzer PEMMA-102-02 (JSC “SELMI”, Sumy, Ukraine). The range of the accelerating voltage change was 0.2–40 keV, the range of magnification change was 10–300,000, and the resolution was no more than 5.0 nm.

The crystal structure of the samples was analyzed by X-ray diffractometry (XRD), for which a DRON-4-07 X-ray Diffractometer (JSC «Bourevestnik», Saint Petersburg, Russia) was used. Irradiating lamps with a copper anode and Ni-filter were used. Investigations were carried out in the range of 2Θ from 4 to 100°.

## 3. Results and Discussion

### 3.1. Metallization of Activated Polypropylene Waste

**Figure 3:**
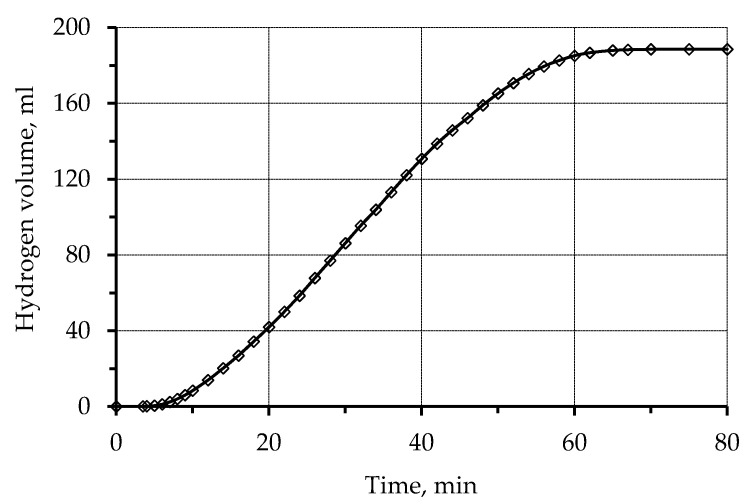
Kinetics of hydrogen release during copper plating of the activated surface of polypropylene waste. Concentration (mmol/L): CuSO_4_-60; EDTA-Na2–67; NaOH-375; formaldehyde 366.

**Figure 4:**
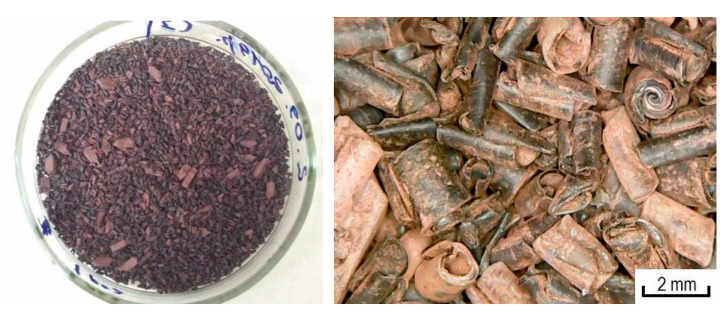
Photomicrographs of copper polypropylene waste.

The use of activated polypropylene waste showed that copper ions are reduced in a solution of chemical metallization (Figure 3). In this case, the reduction of copper occurs on the activated polypropylene surface, which can be seen based on a photomicrograph of copper polypropylene waste (Figure 4). Thus, it allowed establishing the fundamental possibility of reduction of copper ions on the activated polypropylene surface to obtain metalized polymeric raw materials.

Analysis of the photomicrograph of polypropylene waste (Figure 1a and Figure 4) obtained as a result of mechanical processing of car interior panels shows that this polymer raw material is very uneven, both in shape and size. This in turn will create significant difficulties in explaining the results of the study of the process of chemical metallization of such raw materials and the choice of the necessary parameters for its implementation.

Since the process of metallization of the polymer surface in the proposed technology is crucial for obtaining metalized raw materials of the required quality, it was decided to use model polymeric raw materials for studying the kinetics of reduction of copper ions. The use of model polymeric raw materials will allow a more thorough study of the process of metallization of the polypropylene surface, in particular, in relation to the influence of the polymer surface area on the metallization process. Moplen HF501N polypropylene, which is characterized by a wide fractional composition and consists of spherical particles, was chosen as a model polymer. This will allow a thorough analysis of the influence of the area of the activated polymer surface on the patterns of obtaining metalized polymer raw materials.

### 3.2. Sieve Analysis

**Figure 5:**
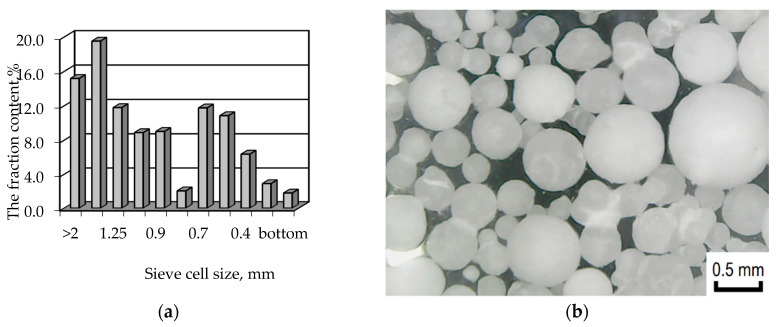
Particle size distribution (**a**) and photomicrographs (**b**) of polypropylene brand Moplen HF501N.

The sieve analysis showed that the polypropylene brand Moplen HF501N mainly consists of a fraction with a particle size greater than 1 mm (Figure 5), with a significant content of polymer fractions with a particle size of about 0.6 mm.

To study the effect of the polymer surface area on metallization regularity, the following fractions were selected from the sieves: 0.5, 0.7, 1.0, 1.6, which most fully characterize this polymeric raw material.

Since the same mass of activated polypropylene (5 grams) was used in all cases for the study of metallization kinetics, there is a direct relationship between the particle size of a certain fraction of polypropylene and the area of the activated surface in contact with a chemical copper plating solution.

The calculation results of the dependence of the surface area of the investigated polypropylene mass on the particle size of a certain fraction are given in Table 1.

### 3.3. Metallization of Activated Polypropylene Brand Moplen HF501N

Obtaining high-tech metal-containing composites based on polypropylene primarily requires information about the patterns of metal layer formation on the activated polymer surface. The study method of the kinetics of metallization of the activated polymer surface in solutions of chemical copper plating, which was used here, is based on the following kinetic equation:

(8) $$2{\mathrm{CH}}_{2}O+{\mathrm{Cu}}^{2+}+4{\mathrm{OH}}^{-}\to \mathrm{Cu}↓+H_{2}↑+2{\mathrm{HCOO}}^{-}+2H_{2}O$$

and takes into account only the amount of copper that is reduced by reaction with formaldehyde. According to this equation, the reagents for the reduction of copper ions are formalin and sodium hydroxide.

Another competitive reaction of copper ion reduction, which takes place in the proposed method, occurs without the release of hydrogen and is an exchange reaction with zinc:

(9) $${\mathrm{CuSO}}_{4}+\mathrm{Zn}\to \mathrm{Cu}↓+{\mathrm{ZnSO}}_{4},$$

This reaction is designed to create conditions for the autocatalytic reaction of copper reduction with formaldehyde and occurs only at the initial stage of the process.

Thus, the kinetic curves obtained in the process of the volumetric metallization study show only the amount of copper recovered by the reaction with formaldehyde.

**Figure 6:**
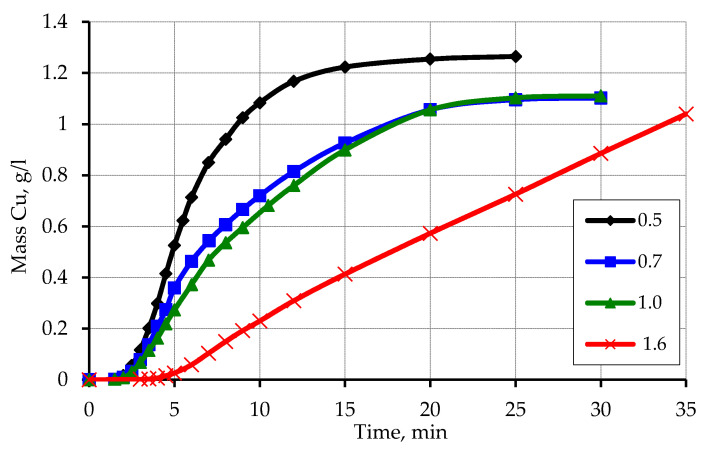
Kinetic curves of reduction of copper ions on the activated surface of polypropylene depending on the particle size. Concentration (mmol/L): CuSO_4_–48; EDTA-Na_2_–67; NaOH-250; formaldehyde–366.

The obtained kinetic curves of copper ion reduction on zinc-activated polypropylene depending on the particle size (Figure 6) showed a significant dependence of the rate of copper ion reduction on the particle size of polypropylene.

In the case of a fraction larger than 1.6 mm, the reduction rate of copper ions is the lowest and is characterized by the largest induction period. At this time, the fraction of polypropylene from a sieve with a cell size of 0.5 mm is characterized by the highest rate of reduction of copper ions and the smallest induction period. The polymer fractions obtained from sieves with cell sizes of 0.7 and 1.0 mm occupy an intermediate position and are close in value. This feature can be explained by the different contact area of the zinc-activated polymer surface, which interacts with a solution of chemical metallization and on which the reduction of copper ions occurs. The increase in the reduction rate of copper ions in the case of a decrease in the particle size of polypropylene can be explained by the increase in the area of the activated surface. An increase in the area in contact with the chemical precipitation solution is equal to an increase in the concentration of the activator metal.

It should also be noted that the unexpected effect of the amount of activator metal on the amount of hydrogen released during the reaction with formaldehyde (which was used to calculate the mass of reduced copper). It can be assumed that for smaller fractions of polypropylene, the amount of released hydrogen should be smaller compared to fractions with larger particle size due to a larger amount of activator metal on their surface. A significant amount of activator metal will promote a deeper exchange reaction with zinc, which takes place without the release of hydrogen. However, there is an opposite dependence: at a zinc content of 6.5 wt.% in the fraction of 0.5, the amount of reduced copper compared to the fraction of 1.6, for which the zinc content is 1.8 wt.%, is greater.

To increase the flexibility and efficiency of the process of polypropylene metallization, a series of studies were conducted on the effect of changes in the concentration of reagents on the rate of copper ions reduction.

**Figure 7:**
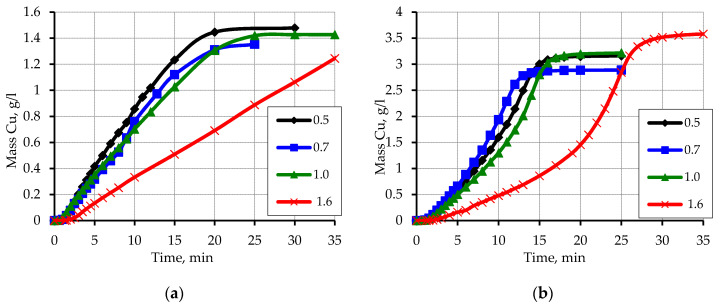
Kinetic curves of copper ion reduction on the activated surface of polypropylene depending on the particle size. Concentration (mmol/L): (**a**) CuSO_4_–48; EDTA-Na_2_–67; NaOH–560; formaldehyde–366. (**b**) CuSO_4_–80; EDTA-Na_2_–67; NaOH–560; formaldehyde–366.

An increase in the concentration of sodium hydroxide to 0.56 mol/L affects some increase in the amount of reduced copper as a result of interaction with formaldehyde (Figure 7). In all cases, at a NaOH concentration of 0.56 mol/L, the reaction of copper ions reduction occurs to the end, as evidenced by the complete discoloration of the solution after metallization compared with the blue initial solution. Moreover, the discoloration of the solution occurs at the time of cessation of hydrogen evolution, which indicates that in the final stages of the reaction of copper ion reduction occurs due to interaction with formaldehyde.

**Figure 8:**
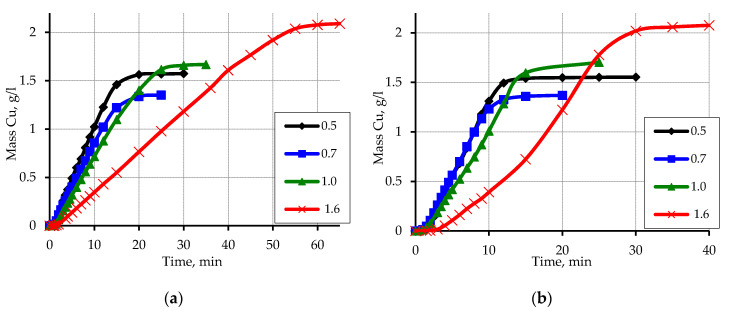
Kinetic curves of copper ion reduction on the activated surface of polypropylene depending on the particle size. Concentration (mmol/L): (**a**) EDTA-Na_2_–54; CuSO_4_–48; NaOH–560; formaldehyde–366. (**b**) EDTA-Na_2_–47; CuSO_4_–48; NaOH–560; formaldehyde–366.

Another factor that has a significant impact on the kinetics of copper ions reduction is the concentration of EDTA-Na_2_. The decrease in the concentration of the complexing agent affects the increase in the rate of copper ion reduction. This is especially true for solutions with a concentration of EDTA-Na_2_ 47 mmol/L (Figure 8b). This can be explained by a certain loss of stability of chemical precipitation solutions, which increases the rate of the reduction reaction [33].

**Figure 9:**
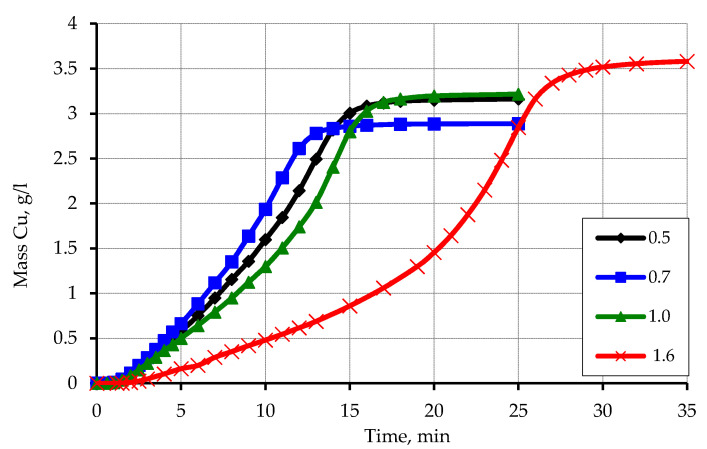
Kinetic curves of copper ion reduction on the activated surface of polypropylene depending on the particle size. Concentration (mmol/L): CuSO_4_–80; EDTA-Na_2_–67; NaOH–560; formaldehyde–366.

When studying the effect of CuSO_4_, it was found that increasing its concentration to 80 mmol/L most significantly affects the rate of copper ion reduction and its amount (Figure 9). A significant acceleration of the reduction reaction of copper ions with formaldehyde in the case of polypropylene fraction 1.6 in the range of 20–25 min, and less noticeable-for the fraction of 1.0 in the range of 12–15 min should also be noted.

Thus, based on research, we can conclude that the factors that have the greatest impact on the copper plating process are the concentrations CuSO_4_, NaOH i EDTA-Na_2_.

At the same time, despite the high speed of the process of copper ion reduction, especially for fractions of polypropylene with small particle size, there is low efficiency of the metallization of the activated polymer surface (Table 2).

**Figure 10:**
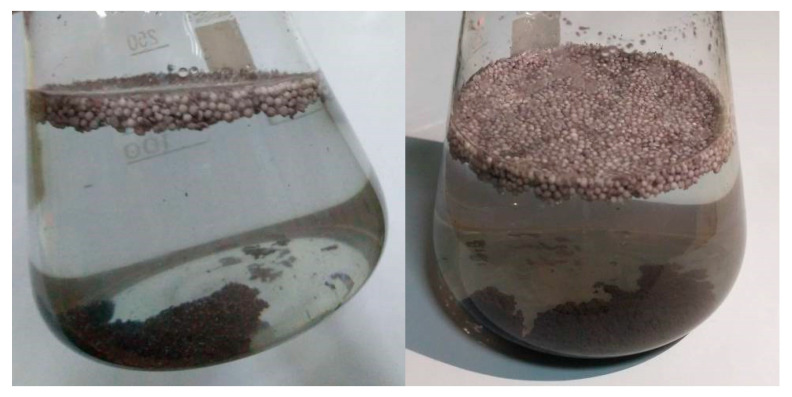
Photograph of the chemical precipitation solution after metallization.

Low efficiency is manifested in the formation of a significant amount of sediment, which consists of reduced copper, which is not connected with the polymer surface in any way (Figure 10).

The formation of a large amount of sediment can be explained by the weak interaction of a significant part of the activator metal with the polypropylene surface, which under conditions of intense mixing leads to washing the metal away from the polymer surface. The presence of an activator metal that is not bound to the polymer surface in the chemical precipitation solution leads to copper ions reduction on zinc particles, which reduces the efficiency of copper plating of the polypropylene surface.

To increase the efficiency of the metallization process, the possibility of reducing the amount of Zn that is washed away during metallization, which causes the copper ions reduction not on the surface of polypropylene, but in the volume of the solution with subsequent precipitation. To do this, the activated polypropylene was washed with water before chemical metallization. This allowed to separate weakly fixed zinc particles and to obtain a fundamentally new activated raw material (Table 3).

**Figure 11:**
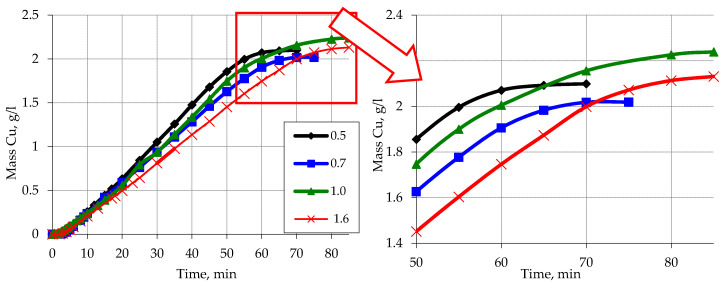
Kinetic curves of copper ion reduction on the activated and washed surface of polypropylene depending on the particle size. Concentration (mmol/L): NaOH–560; CuSO_4_–48; EDTA-Na_2_–67; formaldehyde–366.

The obtained kinetic curves of copper ion reduction on the activated polymer surface, which is devoid of weakly fixed particles, show that in this case there is a slightly different nature of metallization (Figure 11). In this case the dependence of the process of metallization of polypropylene on the sizes of particles, as well as on the area of the activated polymeric surface is less. That proves the participation in the reaction of copper ions reduction of free (washed from the polymer surface) Zn particles, which largely determine the features of copper ions reduction in the case of not washed activated polymer raw material.

The amount of copper that is reduced by the reaction with formaldehyde is higher when using washed activated polypropylene. What is more, the effect of the size of the polypropylene fractions is manifested only in a slightly higher rate of completion of the reaction for smaller fractions, as well as a slightly smaller amount of reduced copper as a result of interaction with formaldehyde.

**Figure 12:**
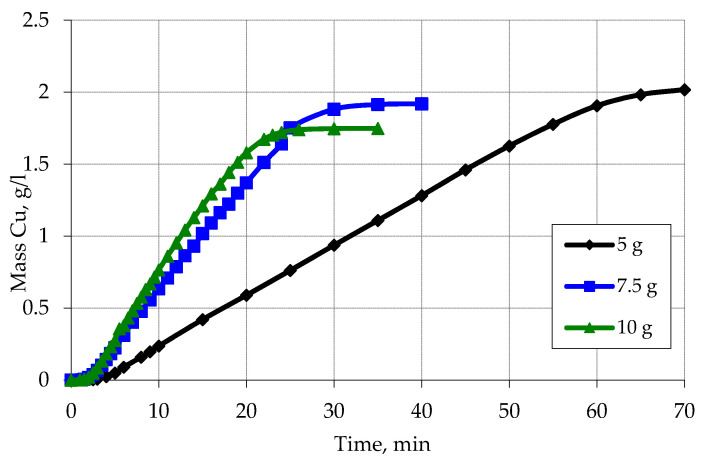
Kinetic curves of reduction of copper ions on the activated surface of polypropylene for the fraction of 0.7 depending on the degree of its loading. Concentration (mmol/L): CuSO_4_–48; NaOH–560; EDTA-Na_2_–67; formaldehyde–366.

In addition to the change in the concentration of components of chemical metallization solutions, the influence of the degree of loading of activated polypropylene raw materials on the regularity of the copper plating process was also investigated (Figure 12). Washed activated polypropylene was used for this.

In this case, the decisive factor that influences the appearance of the kinetic curves is the area of the activated surface in contact with the chemical precipitation solution. Increasing the degree of loading (increasing the contact area) affects the increase in the rate of metallization, as well as in the decrease in the amount of copper, which is reduced as a result of interaction with formaldehyde with the release of hydrogen.

The use of washed activated polypropylene to obtain metalized polymeric raw materials showed high efficiency of this solution (Table 4). The efficiency values of copper plating of such raw materials are significantly higher compared to the results of Table 2.

**Figure 13:**
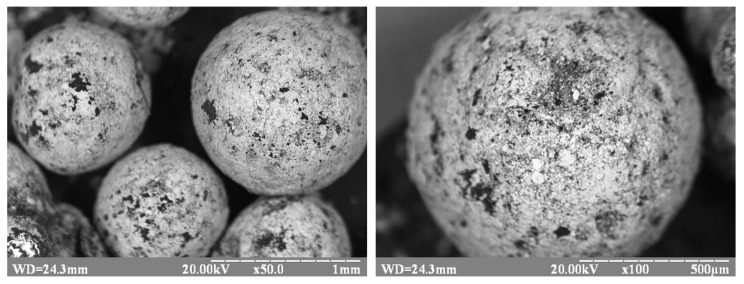
Micrograph of the surface of copper polypropylene obtained in the mode of contrast by the average atomic number (light area-copper).

**Figure 14:**
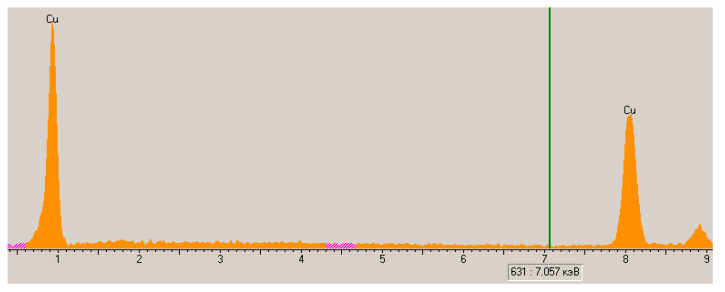
The spectrum of characteristic X-rays of the surface of copper polypropylene.

Studies using a scanning electron microscope in contrast mode and identification of the spectrum of characteristic X-rays of the metalized surface show that the obtained metalized polypropylene is characterized by the formation of a copper coating on polymer particles with a high degree of surface coverage (Figure 13 and Figure 14).

**Figure 15:**
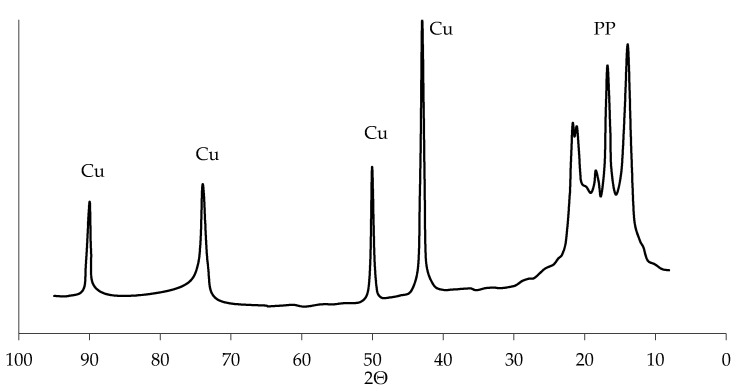
X-ray film of copper coating obtained by chemical recovery.

It should also be noted that the spectra of the characteristic X-rays of the surface of copper polypropylene do not have peaks corresponding to oxygen. This allows us to conclude that there are no copper oxides in the coating, which was also noted by other researchers who received a copper coating in EDTA-Na_2_ solutions of chemical metallization [35]. The absence of oxides in the copper coating is also indicated by the diffraction pattern of copper polypropylene with no peaks on that can be attributed to copper oxides (CuO (35°, 38°, 61°) and Cu_2_O (29°, 36°, 61°)). There are only peaks that are responsible for the crystal structure of polypropylene and copper on the diffraction pattern (Figure 15).

The use of a model polymer provided a convenient study of the process of chemical metallization of the activated polypropylene surface. This guarantees a controlled and effective influence on this process and will allow you to choose the most optimal compositions of solutions to obtain metalized polymeric raw materials of the required quality with a controlled and predetermined metal content.

**Figure 16:**
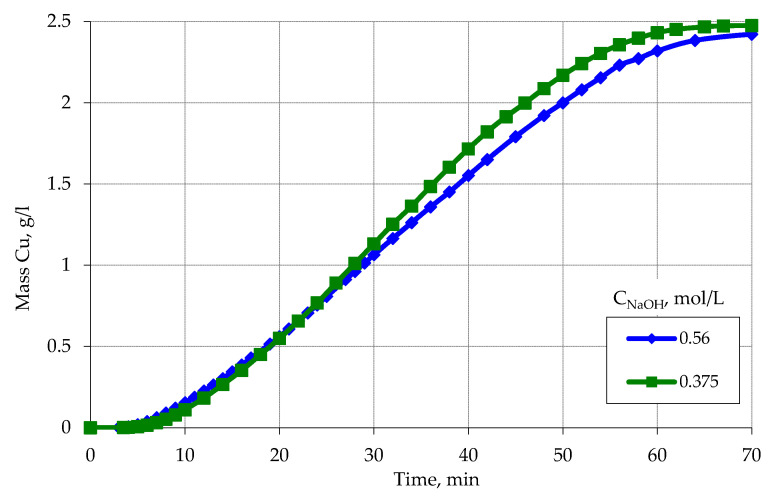
Kinetic curves of copper ion reduction on the activated surface of polypropylene waste. Concentration (mmol/L): CuSO_4_–60; EDTA-Na_2_–67; formaldehyde–366.

The recommendations on carrying out the process of copper plating of the activated waste of polypropylene can be formed based on the conducted research. The metal content of this waste can be adjusted by both the composition of the solution and the degree of loading of secondary raw materials. Also, to significantly increase the copper content in polymer waste, a method of multiple metallization of one polymer raw material can be recommended. In the case of using the method of re-metallization the layer of copper already formed on the surface of polypropylene is a very effective activator of the reduction process. Figure 16 shows the kinetic curves of copper ions reduction on the activated surface of polypropylene waste, which are similar to the recovery curves of copper ions on the model polymer.

### 3.4. Properties of Copper waste Polypropylene

**Figure 17:**
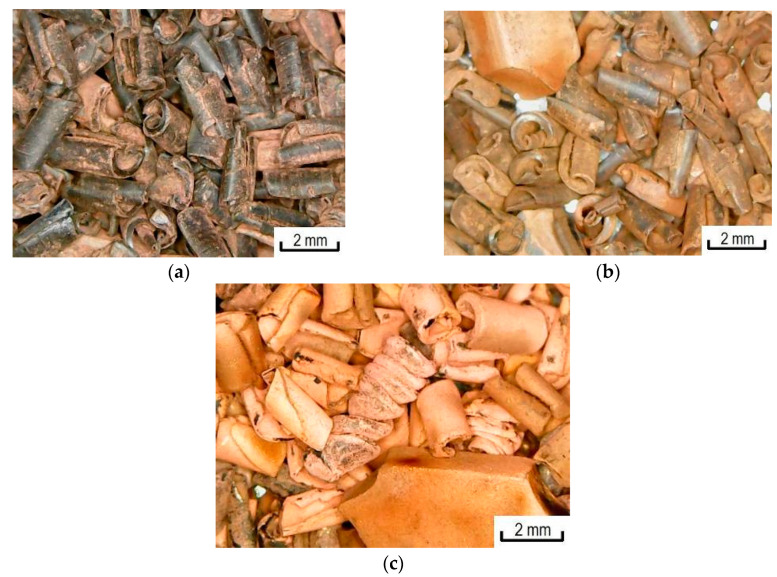
Photomicrographs of polypropylene waste. Copper content: (**a**) 5 wt.%; (**b**) 15 wt.%; (**c**) 20 wt.%.

Photomicrographs of copper samples of polypropylene waste (Figure 17) with different amounts of copper show that the increase in the amount of metal affects the degree of metal coating of the polymer surface. In the case of samples with a metal content of 20 wt.%. The entire polymer surface is almost completely covered with metal.

**Figure 18:**
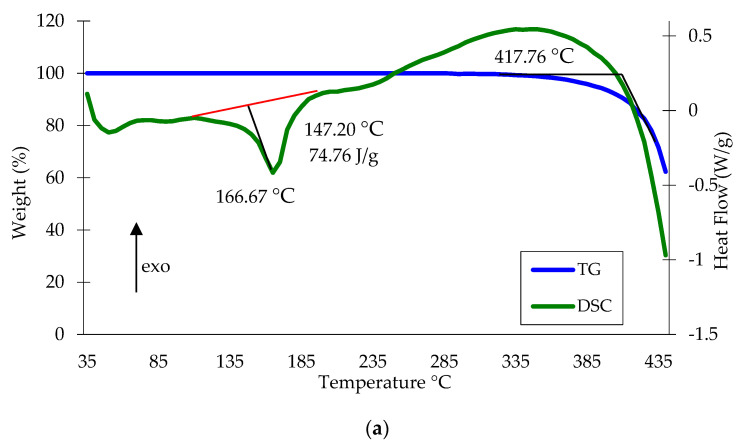

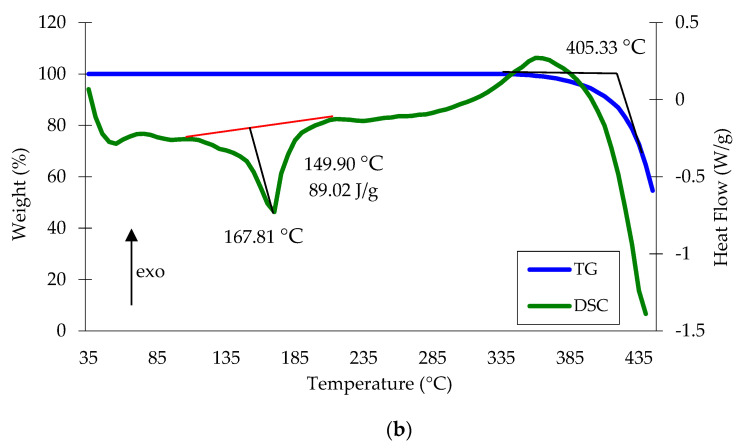
DSC and TG curves of polypropylene waste (**a**) and metalized polypropylene waste (**b**) (copper content 5 wt.%).

The study of raw materials (polypropylene waste) and the obtained metalized polypropylene waste by DSC showed a certain effect of metal on the magnitude of thermal effects (Figure 18).

The presence of copper in the composite has almost no effect on the temperature of the maximum end effect caused by the melting of the crystalline phase of polypropylene; however, its value increases slightly. It can be noted that more significant influence of copper presence on thermal effects in the field of high temperatures. In this case, the peak on the curve of DSC is more pronounced. Besides, the temperature on the curve of thermogravimetric analysis, which corresponds to the maximum rate of mass loss for the metalized polymer is 12 °C lower compared to non-metalized waste, which may indicate their better thermal conductivity.

**Figure 19:**
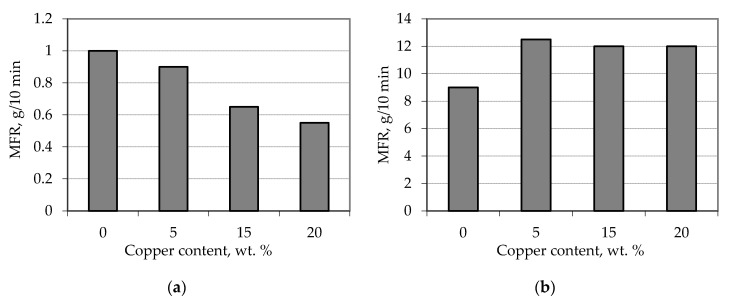
Dependence of MFR of metal-filled polypropylene composites on copper content. (**a**) T = 190 °C, P = 10 kg; (**b**) T = 230 °C, P = 5 kg.

Samples of copper-plated waste polypropylene were characterized by value MFR (Melt Flow Rate) (Figure 19).

The rheological properties of polymer composites depend on the interaction between the filler and the polymer matrix. This interaction becomes possible as a result of adsorption on the surface of the filler of macromolecules resulting in the formation of polymer shell of a certain thickness around the filler particle. There is also a temperature dependence of the thickness of the adsorption layer of macromolecules on the filler particles. These phenomena can explain the obtained results of MFR measurements at different temperatures.

Measurement of MFR at a temperature of 190 °C showed that the increase in copper content affects the increase in melt viscosity (decrease in MFR) (Figure 19a). It can be assumed that in this case, the filler particles (copper) are covered with an adsorption layer of polymer resulting in an effective increase in their volume. As the particle and the associated polymer layer move together, the viscosity increases.

The amount of copper has the opposite effect on the MFR value at a temperature of 230 °C (Figure 19b). In this case, the decrease in viscosity can be explained by the effect of both temperature and shear rate. As the temperature increases, the thickness of the adsorption layer decreases and the mobility of macromolecules increases. This, as well as the increase in the shear rate affects the fact that the polymer melt does not form a stronger structure compared to the unfilled polymer. The interaction between the polymer macromolecules and the filler particles is not strong enough and does not lead to the formation of a stronger network.

Samples obtained from copper-plated waste polypropylene by injection molding showed that the influence of the amount of metal on the strength properties of the obtained metal-filled composites is insignificant and is manifested in some increase in tensile strength and reduced ductility (Table 5).

The high strength properties of the obtained composited can be explained from the standpoint of forming a homogenous structure. Due to the use of pre-metalized polypropylene waste in the process of its injection molding processing, uniform distribution of metal filler in polymer matrix occurs, which provides high strength properties.

## 4. Conclusions

Thus, it can be claimed that the proposed method of introducing a metal filler into the polymer matrix with the help of a chemical metallization of the surface of polymer raw material is effective and can be used to obtain high-tech metal-filled polymer composites, including those based on waste polymeric materials. The formation of a metal shell on the polymer surface, which is destroyed during the melting of the polymer, ensures easy introduction and uniform distribution of the metal over the volume of the material.

Studies on the influence of concentration factors on the process of chemical copper plating of zinc-activated polypropylene allow us to identify the main factors influencing the process of obtaining metalized polypropylene. The amount of reduced copper on the activated polypropylene surface can be adjusted by changing the concentration of CuSO_4_, EDTA-Na_2_, and NaOH, as well as the degree of loading. Such information will allow us to obtain polymeric raw materials of the required quality and to control the metal content in the final metal-filled composite at the stage of obtaining metalized raw materials. The introduction of metal into the polymer matrix in the form of a metal coating formed on the polymer surface guarantees the production of metal-containing polymer composites, which are characterized by uniform distribution of metal in the polymer matrix and high technological and operational properties. Obtaining such metal-filled composites will occur directly during the processing of metalized polymeric raw materials. The properties of the obtained materials can be predicted, as well as changed by the metal content at the stage of the metallization of the polymer surface.

## Figures and Tables

**Table 1:** The dependence of the surface area of 5 grams of polypropylene on the particle size.

Sieve Cell Size (mm)	The Average Particle Diameter of the PP on the Sieve (mm)	The Surface Area of Polypropylene (cm^2^)
1.6	1.8	185
1.0	1.125	290
0.7	0.75	440
0.5	0.6	550

**Table 2:** The efficiency of metallization of activated polypropylene.

PP Fraction on a Sieve	Concentration (mol/L)		Metallization Efficiency (%)
	NaOH	CuSO_4_	
0.7	0.25	48	27.0
0.7	0.56	48	11.5
0.7	0.56	80	34.8
0.5	1.68	144	35.8

**Table 3:** The content of the activator metal on the polypropylene surface.

PP Fraction on a Sieve	0.5	1.0	1.6
The content of Zn (wt.%)	Activated polypropylene		
	6.6	5.4	1.8
	Washed activated polypropylene		
	0.9	0.5	0.6

**Table 4:** Metallization efficiency of washed activated polypropylene.

PP Fraction on a Sieve	Concentration (mol/L)		Metallization Efficiency (%)
	NaOH	CuSO_4_	
**0.7**	0.56	48	98.2

**Table 5:** Physicomechanical properties of metal-filled composites.

The Copper Content (% mass)	Ultimate Tensile Strength (MPa)	Strain at Strength (%)	Tensile Stress at Break (MPa)	Strain at Break (%)
0	57.5	30	51.4	37
5	57.2	30	48.7	35
15	57.5	28	55.1	32
20	-	-	55.0	32
